# Practical guidance for planning resources required to support publicly-funded adaptive clinical trials

**DOI:** 10.1186/s12916-022-02445-7

**Published:** 2022-08-10

**Authors:** James M. S. Wason, Munyaradzi Dimairo, Katie Biggs, Sarah Bowden, Julia Brown, Laura Flight, Jamie Hall, Thomas Jaki, Rachel Lowe, Philip Pallmann, Mark A. Pilling, Claire Snowdon, Matthew R. Sydes, Sofía S. Villar, Christopher J. Weir, Nina Wilson, Christina Yap, Helen Hancock, Rebecca Maier

**Affiliations:** 1 Biostatistics Research Group, Population Health Sciences Institute, Newcastle University, Newcastle upon Tyne, UK; 2 School of Health and Related Research, Clinical Trials Research Unit, University of Sheffield, Sheffield, UK; 3 Cancer Research UK Clinical Trials Unit (CRCTU), University of Birmingham, Birmingham, UK; 4 Cancer Research UK CTU, University of Leeds, Leeds, UK; 5 School of Health and Related Research, Health Economics and Decision Science, University of Sheffield, Sheffield, UK; 6 MRC Biostatistics Unit, University of Cambridge, Cambridge, UK; 7 Department of Mathematics and Statistics, Lancaster University, Lancaster, UK; 8 Centre for Trials Research, Cardiff University, Cardiff, UK; 9 Department of Public Health and Primary Care, University of Cambridge, Cambridge, UK; 10 The Institute of Cancer Research Clinical Trials & Statistics Unit, London, UK; 11 MRC Clinical Trials Unit at UCL, London, UK; 12 Edinburgh Clinical Trials Unit, Usher Institute, University of Edinburgh, Edinburgh, UK; 13 Newcastle Clinical Trials Unit, Newcastle University, Newcastle upon Tyne, UK

**Keywords:** Adaptive designs, Adaptive clinical trials, Clinical trials, Efficiency, Resource requirements, Trial coordination

## Abstract

Adaptive designs are a class of methods for improving efficiency and patient benefit of clinical trials. Although their use has increased in recent years, research suggests they are not used in many situations where they have potential to bring benefit. One barrier to their more widespread use is a lack of understanding about how the choice to use an adaptive design, rather than a traditional design, affects resources (staff and non-staff) required to set-up, conduct and report a trial. The Costing Adaptive Trials project investigated this issue using quantitative and qualitative research amongst UK Clinical Trials Units. Here, we present guidance that is informed by our research, on considering the appropriate resourcing of adaptive trials. We outline a five-step process to estimate the resources required and provide an accompanying costing tool. The process involves understanding the tasks required to undertake a trial, and how the adaptive design affects them. We identify barriers in the publicly funded landscape and provide recommendations to trial funders that would address them. Although our guidance and recommendations are most relevant to UK non-commercial trials, many aspects are relevant more widely.

**Supplementary Information:**

The online version contains supplementary material available at 10.1186/s12916-022-02445-7.

## Background

Clinical trials are a vital part of improving the treatment and care of patients. Due to the increasing costs of trials [1, 2] and the need to answer important research questions as rapidly and robustly as possible, new trial methods that can increase operational and statistical efficiency are of great interest. Adaptive trial designs [3] are one such class of methods; they provide pre-planned opportunities to use accumulating trial participant outcome data to make changes to the course of the trial, whilst ensuring the statistical properties of the trial remain intact and results credible. Adaptive designs (ADs) have different features that can, for example, (1) improve the statistical power of the trial; (2) reduce the time taken and the number of participants required to evaluate treatments, thus potentially saving money and other resources; and (3) reduce exposure of trial participants to insufficiently effective, or more harmful, treatments by stopping recruitment to them early [4]. ADs are typically more statistically and operationally complex than traditional trials and require high-quality interim analyses undertaken (including implementation of decisions) rapidly; they may therefore require higher levels of (and exact timing of) effort, resources and expertise to design, set-up, deliver, analyse and report. The sample size and study length of an adaptive trial are often unknown at the outset, which can further complicate their resourcing. There has been little guidance aimed at non-commercial organisations and researchers who conduct clinical trials on appropriate resourcing of adaptive trials. If adaptive trials are inadequately resourced, their advantages may be compromised, leading to increased risk of operational or statistical biases [3, 5, 6].

### The costing adaptive trials project

The Costing Adaptive Trials (CAT) project investigated the additional resources, as compared to similar non-ADs, required to support effective adaptive trials. Full details of the methods and results are reported in Wilson et al. [7]. Briefly, this research was undertaken in the UK in 2020 through a mock costing exercise. Research staff in seven academic UK Clinical Research Collaboration (CRC) registered Clinical Trials Units (CTUs) provided the staff and non-staff financial costs that they estimated were required to support an adaptive version of a trial, and a non-adaptive version of the same trial. This was undertaken for five different trial scenarios covering different types of ADs based on real trials run in the UK (see Additional file 1). The level of practical experience of the designs varied across the CTUs. The mock costing exercise was followed by a qualitative research component to understand the factors influencing the estimated resource requirements and differences between the non-adaptive and adaptive trial designs, as well as between CTUs.

Results demonstrated wide variability in the staff and non-staff resources anticipated across scenarios and CTUs, dependent, for example, on availability of core infrastructure programme funding or in-house IT systems. On average, there was a modest increase (2–4%) in resources anticipated for the AD, compared to the non-AD, within each scenario. This is consistent with comparisons using alternative methods [8]. The highest percentage increase was for statistical staff, followed by data management staff. There was inconsistency in whether additional resources for trial management staff were required across CTUs.

An important objective of the CAT project was to use results from the research to develop guidance for non-commercial organisations and researchers who design, plan, coordinate and deliver clinical trials. Here, we outline a five-step approach to aid, and potentially shorten, the time-consuming planning of adequate resourcing of adaptive trials (both staff time and non-staff costs). This approach was informed from our CAT research results that focussed on CTU resources (excluding per-patient costs). We did not explore other research costs such as intervention supply, or other methodological groups that may be involved in a trial, for example, health economists or researchers using qualitative methods. Thus, these are not fully considered in this guidance.

**Fig. 1:**
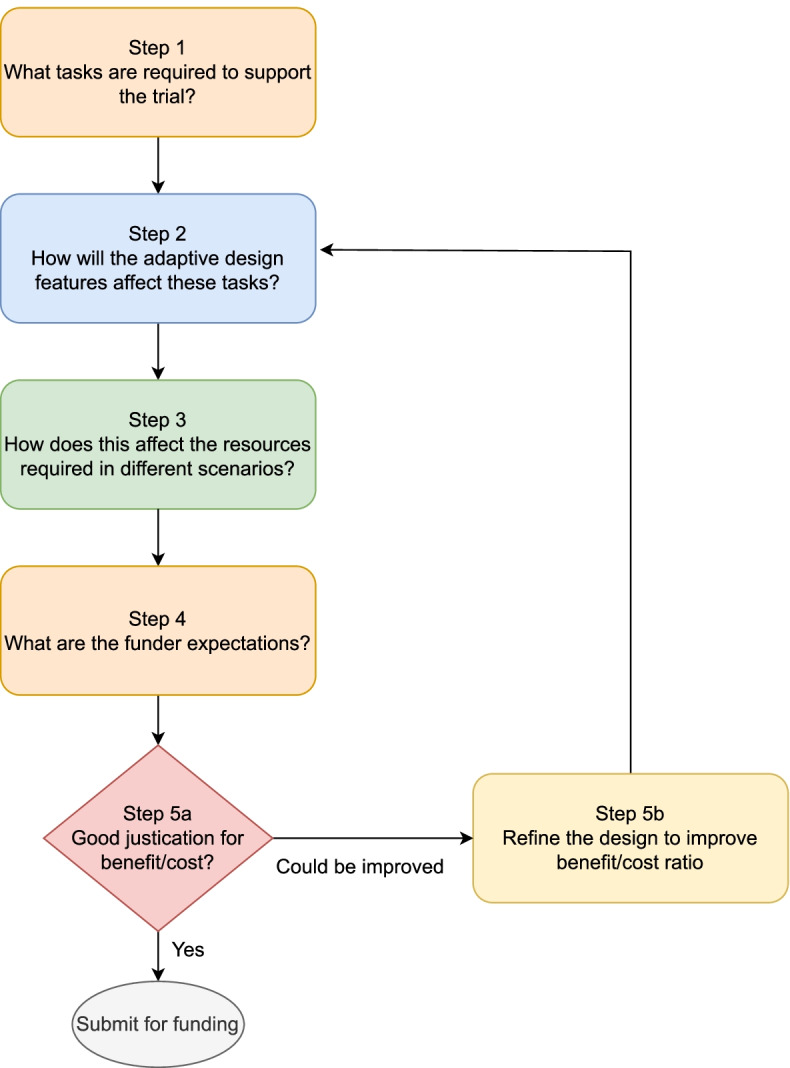
Outline of process for considering and justifying resources for an adaptive design

The process is shown in Fig. 1, with each step described in further detail below. It is predominantly aimed at academic organisations and researchers running clinical trials, but also may be relevant to other organisations and funders. Specific recommendations to funders are provided in the ‘ Guidance for funders’ section. Although we focus on ADs, the process may be useful for other innovative designs such as master protocols [9] and seamless designs (e.g. phase II/III) which are often adaptive, but not always.

### Step 1 – What tasks are required to support the trial?

We start with the assumption that there is a proposal for a clinical trial, following the PICOS (Participants, Intervention, Comparator, Outcomes, Statistical analysis) framework [10]. Before following the process here, it is recommended to consider first whether an AD is suitable. For example, if the outcome measure on which the adaptations are based is not observed sufficiently quickly, then an AD is unlikely to provide improved utility [11].

Assuming an AD is suitable, it is helpful to first map out a recruitment strategy (including estimated sample size, number of sites, length of recruitment), a Gantt chart and the tasks that are required to support a clinical trial, regardless of whether an AD is used. We show some major tasks common to trials in Table 1 and how ADs impact on staff and non-staff resources in Table 2.

**Table 1:** Major tasks required to run a clinical trial and how adaptive designs may affect them

Major tasks	How might an adaptive design affect the task?	Potential resource implications
Development of trial design, protocol and trial materials including SOPs	More scenarios to plan, possibly involving pre-trial simulation studies, and more milestones	Additional statistical and trial management staff resource
Regulatory, ethical and governance applications	Increased complexity in communicating the design in applications, greater chance revisions may be required, more complex contracting	Additional trial management, statistical, and administrative staff resource
Database set-up and maintenance	Case report forms and database may change during the trial due to adaptations; more complexity and thought needed based on the scenario planning during trial development to enable adaptations to be managed efficiently; more testing required	Additional data management, programmer, statistical and trial management staff resource; higher fees for outsourced services
Randomisation system set up and maintenance	Randomisation method may be bespoke and not implemented in standard systems; randomisation systems may need updating during the trial; more complexity and thought needed based on the scenario planning during trial development to enable adaptations to be managed efficiently; more testing required	Additional data management, programmer, statistical and trial management staff resource; higher fees for outsourced services; cost/time of making changes to randomisation systems if trial changes
Site set-up (and securing service support costs and excess treatment costs)	Contracts may need to reflect variability in expectations of recruitment periods, breaks in recruitment, expectations on data entry and cleaning to enable robust decisions based on timely cleaned and locked data. There may also be variability in excess treatment costs required due to change in dose or sample size. More frequent site training generally required	Additional trial management resource
Data queries and cleaning	Requires more time and ongoing review for cleaning	Additional data management staff and statistical resource
Interim analyses and data monitoring	May require more monitoring at centres or centrally in addition to data cleaning prior to data lock; requires interim statistical analysis plan; requires time for interim statistical analyses	Additional data management, statistical and trial management staff resource
Statistical analysis plan	Requires rigorous upfront development with scenario planning; may involve running extensive simulations to ascertain the design’s operating characteristics	Additional statistical staff resource
Statistical analysis	May require additional staff to protect core team from knowing accumulating, comparative results; may involve more advanced statistical methods (e.g. for point and interval estimation, multiplicity adjustment or Bayesian analysis) and additional programming	Additional statistical staff resource
Close-down	Timing will depend on outcomes of interim analyses	May require more buffer room to allow for uncertainty, especially for smaller institutions

**Table 2:** Resources where adaptive designs increase use

Resource required	Examples of reasons of additional resource
Trial manager time	More complex protocol development More time to create patient information sheets Complexity of design Amendments Additional or more frequent meetings Data cleaning co-ordination for interim analysis Increased site communication and training Additional user testing of updated systems Increased co-ordination (i.e. timing of drug supply) Regulatory interactions Contract negotiations
Statistician time	Simulations of design operating characteristics Protocol development (Interim) SAP development Interim analysis Trial Steering Committee/Data Monitoring Committee Report preparation More complex final analyses Additional “unblinded” statistician Additional quality control statistician Specification of system needs, user testing of systems
Data manager/programmer/information specialist time	Increased set-up resource to prepare for planned adaptations and to build more complex databases/randomisation systems Increased time for complexity of data management plan Data cleaning for interim analysis Database lock for interim analyses Database amendments due to adaptations
Staff with specialist expertise (e.g. senior statisticians/methodologists/trial manager)	Complexity of design Expertise required in adaptive designs Understanding consequences of adaptations
Intervention costs	Extended timelines, and costs for changes in drug manufacture due to an adaptation (e.g. dose changes) Intervention-related data collection costs
Non-staff CTU costs	Training Additional meeting costs Regulatory agency fees for amendments Additional travel costs if on-site monitoring is needed License fees for specialist software
Other	Increase in timelines for adaptive designs due to planned breaks in recruitment due to interim analyses (not in all cases) Increase in resource to handle uncertainty

For each task, it is useful to consider the workload required by broad categories of staff. The broad staff categories identified in the CAT project were trial management, data management and statistics. However, some institutions will have staff that might cross several categories (such as a statistical programmer) or be a cross-cutting role (e.g. quality assurance) and some trials will require staff from other methodological areas such as health economics not considered here. Some tasks will predominantly cause workload for one staff category (e.g. writing a Statistical Analysis Plan will be the responsibility of statistical staff with substantially less input from staff from other categories) whereas others may involve more than one (e.g. setting up and testing the randomisation system).

Some CTUs who took part in CAT described using their own costing template (also known as a budget planning tool [8]) that captured the amount of work or time required, which makes the costing process easier and more transparent. We have provided an example costing tool, implemented in a spreadsheet, in Additional file 2providing the list of tasks mentioned in Tables 1 and 2.

Some resources are also affected by the institution itself. Some CTUs participating in CAT had their own in-house randomisation and clinical data management system (CDMS); others outsourced these tasks to third parties. These differences in available infrastructure will influence staff workload (across categories) as well as non-staff costs required depending on the adaptive features considered. Some institutions had core infrastructure programme funding that will influence staff resources and non-staff costs required.

Once the trial team has a good understanding of the resource required for the proposed non-adaptive study, it is time to progress to Step 2. We would note that in some cases there may be no equivalent non-adaptive study to serve as a baseline, such as a phase I dose-finding trial, in which case this step may be more difficult.

### Step 2 – How will the adaptive design features affect these tasks?

The next step in the process is considering the implications of the AD features (Table 3) on the tasks identified in Step 1 and how this influences the resources required. Clearly, this will depend on the proposed AD and adaptive features or adaptations considered.

**Table 3:** Additional implications of adaptive design features on resource use

Feature of adaptive/innovative design	Considerations for resources
Number of interim analyses	The more interim analyses generally the more additional resources required. Setting up suitable systems or investing in software may reduce this Additional interim analyses may not always provide additional efficiency of the adaptive design [12]
Adaptive randomisation	Using outcome adaptive randomisation [13] may require more complex randomisation systems than non-adaptive or alternative ADs that drop arms for lack of benefit
Multi-arm multi-stage (MAMS) designs	Some approaches such as group-sequential MAMS [14] may have a more variable sample size than alternatives such as drop-the-losers [15]. The more interim analyses and arms there are, the higher the variability in the sample size
Platform trials	A platform trial [16] may have fixed costs for the underlying infrastructure and an additional cost for each arm added in (the latter of which may be lower compared to a new separate trial)
Dose-ranging trials	Changing which doses are allocated to participants may have associated pharmacy costs and also impact on excess treatment costs
Population enrichment	Changes to eligibility Changes to randomisation method/approach (e.g. a stratification factor or minimisation factor may be dropped) Training of site staff to understand implications Changes to PIS

We first consider elements affecting resource requirements that ADs have in common, across the life cycle of a trial: (1) whilst in set-up; (2) during recruitment and follow-up; and (3) at the time of the final analysis and reporting. We then provide some considerations for specific types of ADs.

#### Trial set-up

Trial set-up involves several tasks that may be affected by the AD given adaptive trials are typically more complex than non-adaptive trials, including writing the protocol, discussion with regulators, applying for regulatory and ethical approvals, development of the database, development of contracts with industry partners, design and development of randomisation systems, and setting up recruitment sites. This means an AD protocol may require more effort (including, for example, fully describing the design and its statistical properties, potentially with additional documents summarising simulations) and extra time may be needed for approvals and site set-up. Based on the experience of the authors, ethics committees and (if applicable) regulators may have queries about the design that require clarifications or amendments and resubmission; extra effort may be required to develop patient information sheets (PIS) that explain the design in a clear, concise and accurate way without giving away too much information that could potentially introduce biases in the conduct of the trial.

#### During recruitment and follow-up: conducting interim analyses and implementation of adaptations

A common property of ADs is that they involve one or more interim analyses whilst recruitment is ongoing and, depending on the results, implementing changes.

Interim analyses require high-quality data on the variables to be used to make adaptation decisions in a timely manner. In some cases, this may be a single outcome variable, but other designs might use a range of outcome variables. As an example, the decision-making at the interim analysis in MIDFUT, an adaptive multi-arm multi-stage trial in diabetic foot ulcer [17], involves an efficacy outcome, as well as safety data and early cost-effectiveness. Resources required for ensuring data are as accurate and complete as possible, known as data cleaning, must be done by the time of each interim analysis; any additional burden on site staff, trial managers, and data managers in the time leading up to the interim analyses should be considered.

Interim analyses also require additional statistical resource to undertake the analyses on the cleaned dataset. As well as the time to do this, additional tasks may be required. For example, an interim statistical analysis plan (SAP) would be required to unambiguously lay out the planned analysis methods in advance of data being available. This is a formal requirement for a Clinical Trial of an Investigational Medicinal Product (CTIMP), and may also require a statistician not involved in the design or conduct of the trial to prepare the interim analyses, allowing the Trial Statistician to remain blind [18] to arm allocation; in other circumstances where this is not a formal requirement, it still may be desirable to ensure that trial results do not influence the conduct of the final analysis. The time for any additional statisticians to understand the complexities of the design should also be considered. Statisticians may also be involved in the data cleaning process, such as in identifying outliers to be checked with sites. All of these factors would increase the resources required for statistical staff.

In some cases, experts in other areas might be required for the interim analysis. An example is the STOP-OHSS group sequential trial [19], assessing the clinical and cost-effectiveness of early active management of ovarian hyperstimulation syndrome compared to usual care. One interim analysis for non-binding futility early stopping is planned. A health economist was involved in the trial design by developing a preliminary model that informed the choice of an appropriate interim futility stopping rule. They will develop interim and final Health Economic Analysis Plans (HEAPs) and update the preliminary health economic model at an interim analysis [20].

After an interim analysis is completed, there may be pre-planned changes required to the sample size and trial systems. This may involve: changes to the CDMS and randomisation systems; making substantial amendments to the protocol and other trial documents and associated regulatory approvals [21, 22]; and implementing changes in trial sites. Depending on the nature of the changes required, this may increase resources required for CTU staff, or require increased costs to make changes to any outsourced systems.

#### Final analysis and reporting

Analysis and reporting will be influenced by the AD, adaptive features, and interim decisions made. The CONSORT extension for randomised adaptive trials [23], which provides guidance on clear reporting of all parts of the trial, is a useful resource to consider.

Overall numbers of data queries and the amount of cleaning around the time of the final analysis may be reduced due to having been brought forward to interim analyses. However, statistical analyses for ADs, especially estimation of treatment effects and related quantities such as confidence intervals, can be more complex than for traditional designs—see Robertson et al. [24] for a recent review. Other analyses, such as health economics, might also become more complex to account for the AD [25, 26].

#### Implications of specific features of the designs

The above considerations are applicable generally in ADs, but their impact may be affected by the specific design and its adaptive features. The number of interim analyses will clearly have an impact. The consequences of interim analyses can be split into ‘fixed costs’ and ‘variable costs’. Fixed costs are present regardless of the number of interim analyses; variable costs will increase (although not necessarily linearly) with the number of interim analyses. Examples of fixed costs would include interim SAP development; variable costs would be the statistical resource required for the interim analyses and effects of implementing changes on trial systems.

Bespoke, complex, novel ADs may require more resource increase for set-up and analysis than more commonly used ADs (such as group sequential or sample size re-estimation designs).

### Step 3 – How does this affect the resources required across all possible scenarios?

Once the impact of the AD on the tasks required is mapped out, the next step is to estimate any impact on the resources required. Adaptive trials are often characterised by their flexibility, leading to uncertainty about how they will unfold. Although some ADs have a fixed sample size, most do not. It is important to consider how resources required for tasks will change if adaptations occur.

Different non-commercial organisations may have various approaches to costing which influence this. Here, we have identified some principles that we recommend are considered by those estimating resource requirements and by funders. We first consider the effects on resources that ADs have in general, and then the effects of specific ADs.

#### General resource impacts of ADs

First, the impact of some tasks on staff resources will likely be at different points in the trial and the timings may be hard to predict upfront. Larger organisations, with larger numbers of staff and more experience in running adaptive trials, may handle this workload planning more easily than those with smaller teams and less experience. For example, if an interim analysis requires one month’s work from a second, blinded, statistician this will be easier to accommodate if the institution has many statisticians than if it only has one. Further examples are provided in Table 4 within the context of the Graves-PCD trial.

**Table 4:** Graves-PCD

Graves-PCD (ISRCTN81162400) is an early phase dose-ranging study coordinated by Newcastle CTU. It is testing four doses of daratumumab against placebo for the treatment of severe Graves’ disease, an autoimmune disorder of the thyroid. The final design involves up to 30 participants will be randomised, split into two stages. After 15 participants (3 per arm) have had primary outcome assessed (change in Serum TRAb antibodies from baseline to 12 weeks) an interim analysis will be conducted. Up to two doses of daratumumab and placebo will continue in the second stage, with the selected doses dependent on a three-parameter Emax model [27] fitted to stage 1 outcome data. Prior to the final design being decided, possible considerations for each of the steps are provided below:
*Step 1*: As an early phase CTIMP, substantial regulatory oversight is required regardless of the design used. The trial required regulatory approval and robust procedures including pharmacovigilance and data monitoring. With a low total number of participants, data errors could cause disproportionate impact on data monitoring. As a dose-ranging study, the final analysis will be statistically complex
These factors mean the CTU and statistical resource required is likely to be high even without an AD. Using a template costing tool allows mapping this out
*Step 2*: An AD that allows updating the stage 2 doses based on stage 1 outcome data has several consequences on trial tasks: (1) some set-up tasks such as protocol development, ethical, and regulatory approval, creating PIS, specification and testing of CDMS and randomisation systems might be more complex; (2) an interim SAP would need developing, at least one interim analysis conducted and any adaptations implemented in a timely manner; and (3) the final statistical analysis and reporting of the trial would be more complex
*Step 3*: More statistical time is required for developing an interim SAP and to conduct interim analyses. More data management time is needed to provide clean data in a timely manner for the statistician at interim analyses. At the time, Newcastle CTU was outsourcing CDMS and randomisation to a third party, and the prospect of a more complex design could mean a higher fee for these services and more staff resource required for testing. More trial management time is required for more complex set-up tasks
The AD initially proposed would allow doses post-interim to depend on results up to that point, leading to uncertainties on which doses are needed and implications on pharmacy support. If stage 2 occurred, the randomisation system requires updating. If there was no evidence of dose–response after stage 1, the trial would stop early, and this would influence the resources required to end the trial (lessening them but still requiring some to close and report the trial)
*Step 4*: The trial was being submitted to MRC DPFS [28] for funding. The panel requires clear description of the statistical properties of the trial, meaning initial statistical simulations were required prior to submission. MRC DPFS requires specification of milestones with staged funding, so having the interim analysis completion as a milestone would allow consideration of the costs incurred at different parts of the trial
*Step 5*: After considering the timelines, costs, and statistical properties it was decided that a single interim analysis would provide most of the benefit possible from the approach without additional costs of more interim analyses

Second, some additional costs of running adaptive trials may be shared across other trials. Examples may be the purchase of specialised software, the development of standard operating procedures (SOPs), or staff training. These types of costs are needed for specific aspects of work; their costs may be attributed to a single trial, split across multiple trials (e.g. with costs calculated ‘per use’), or may be met internally in organisations that have core institutional support.

Third, consideration of how ADs impact National Health Service (NHS) Research Costs, Service Support Costs and Treatment Costs may be required. The informed consent process may take longer due to increased trial complexity requiring more site staff time. Treatment costs may vary due to uncertain sample size, increasing complexity in treatment cost negotiations and requiring additional trial management input.

#### Impact of specific ADs

A common AD incorporates pre-specified criteria for early trial stopping due to lack-of-benefit. If the trial is stopped early this will have an impact on the project duration and resources required to recruit participants. This may lead to a reduction in costs compared to the trial continuing, without cost to the statistical properties of the study. We note that stopping for lack-of-benefit does not immediately bring a trial to an end: following up those who have already been enrolled is likely to continue alongside the closure of trial sites and undertaking a final analysis on all outcomes in order to disseminate the results. Therefore, the remaining staff resource required may be reduced but is not removed.

In other ADs, such as multi-arm multi-stage (MAMS) designs, recruitment to the overall trial may not stop early but recruitment to individual arms may. Until an interim analysis is performed it will remain unknown which, and in some cases, how many arms will continue. There are some tasks required for stopping recruitment to an arm, such as: amending the randomisation system; implementing changes to the PIS and at individual sites [21, 22]; and conducting final analyses for closed arms. There may also be an impact on the time needed to recruit remaining participants if the sample size is specified per arm. Early stopping of arms or the trial may lead to a reduction in overall costs required without compromising the trial’s integrity and validity.

Other ADs, like sample size re-estimation, may potentially increase the target sample size, and therefore trial duration. The change in resources required in this circumstance will depend on how this influences the tasks required. All CTUs who took part in the CAT mock costing exercise provided estimated resources based on the maximum target sample size, and the time needed to recruit to this. This is useful to inform the maximum resource needs. However, one must consider all potential scenarios that can occur, as well as the likelihood of each scenario, and how this will influence the resources required by the trial. In the more theoretic ADs literature, it has been a rule of thumb that quantities such as the ‘average sample size’ (i.e. the sample sizes associated with all possible scenarios, weighted by probabilities of each scenario occurring) can be a good representation of the efficiency of an AD. These metrics are based on multiple hypothetical trials and are not necessarily helpful for the specific trial being costed. They also do not take into account the fixed costs of starting and stopping the trial and the need to have funds available to cover all possible scenarios.

For designs that may lead to a high variation in the resources required, the host institution must carefully consider the implications of this. As in Step 2, larger organisations with many projects and staff may be better able to deal with uncertainty than smaller organisations.

It is important to remember that uncertainty is not unique to ADs. In most trials, there is uncertainty around feasibility aspects such as recruitment rates, and whether specified milestones will be met (e.g. as monitored in the internal pilot phase of a trial [29]). Most non-adaptive trials can also be stopped early, e.g. due to safety concerns.

### Step 4 – What are the funder expectations?

Currently, most funders of academic or public sector trials (in the UK) provide limited flexibility in specifying the costs requested in a funding application. A single figure is required which is considered by the funding panel deciding on whether there is value for money provided by the research. This figure would generally be the maximum required by the trial. If an adaptive trial is highly variable in its cost (e.g. depending on how many arms stop early, or whether the recruitment target increases following a sample size re-estimation) then this maximum may make the research look expensive, but would minimise the risk of needing to return to a funder for additional resources to complete a trial, and also minimise the risk of an inconclusive trial result. In the CAT project, we found that CTUs presented this maximum amount to ensure the research could be delivered in the ‘worst-case scenario’ that requires the maximum sample size.

There should be opportunities to insert more details in the application form to provide an estimate of cost savings if a particular change happens. Most funders have a justification of costs section, which would allow including estimation of cost savings in certain scenarios. In addition, some funders of trials (e.g. the Medical Research Council (MRC) Developmental Pathway Funding Scheme, DPFS [28]) currently require projects to be split into milestones, each with an associated cost. This may allow better specification of the uncertainty in the cost of an AD if milestones are linked to interim analyses and encourage organisations to plan ahead. This last approach has some limitations, such as not allowing alternative paths of milestones depending on the results of an early milestone.

An alternative approach is that funders allow requesting funded extensions of research or variations to contracts. In our experience with UK funders, this has not been commonly encouraged as a way to handle uncertainty in ADs and does not provide certainty of funding in the worst-case scenario. It may, however, be a useful way to handle sample size re-estimation designs or platform trials that can add in new arms.

It is vital to bear in mind the funder requirements and flexibility of the application form when deciding how to best present the uncertainty of the resources required.

### Step 5 – Justifying and refining the design

Considering Steps 2–4 will determine how the design influences the resources required and how this can be communicated to the funder. It is important then to consider how different aspects of the design could be modified to retain most or all of the benefits whilst minimising any statistical or resource issues that arise. This may include considering the number of interim analyses, reconsidering the types of adaptations that are implemented, and the specific decision rules that are specified. As well as considering the resources required by the trial design, it is important to consider the quality of evidence provided by the trial also.

The methods of the value of information analysis (VOIA) may be useful to help consider the benefits and the associated costs of collecting more information to inform (and reduce the uncertainty of) a technology adoption decision [30, 31]. Currently, there is limited use of this approach in practice in the context of ADs [32] as well as non-adaptive designs [33]. However, VOIA methods can be used to quantify the value of non-ADs and alternative ADs allowing the comparison of multiple designs. This can help the research team to refine and justify their chosen design [34, 35]. A clear understanding of the costs of the trial is required for these analyses, which is facilitated by following Steps 1–4.

### Example

Table 4 presents an example of how a funded adaptive trial could have followed the five-step process.

### Guidance for funders

Funders of clinical trials benefit from the appropriate use of ADs as they provide higher efficiency and more robust evidence that ultimately benefits patients. In our experience, some funders have been more encouraging than others in the use of innovative designs [36]. Nevertheless, there are some barriers imposed that may stifle methodological innovation; some of these were raised earlier in this article.

Currently, some aspects of public funding of trials may penalise innovative designs. By only allowing limited space for specifying the requested funding, it may be necessary to focus on the maximum ‘worst-case scenario’ cost which may make the trial look expensive. Having distinct funding panels that typically focus on a particular phase of trial makes getting funding for seamless designs, spanning multiple phases, difficult. Funding agreements that penalise investigators for stopping a trial early because they have answered the research question quicker (i.e. by completely cutting off funding) make some efficient designs unappealing to academic organisations. Furthermore, the additional resources required pre-funding application are usually unfunded, often making organisations reliant on core funding when exploring an adaptive trial design. Funders could promote more use of ADs through making infrastructure awards that would allow further capacity to develop them.

There are some ways in which funders could encourage more innovation by allowing more flexibility in their awards and application processes (Table 5).

**Table 5:** Recommendations to funders to encourage increased appropriate use of innovative designs

We would advise that funders:
1. Develop easily accessible funding schemes that can cover the more intensive development pre-funding work-up period;
2. Recognise that ADs can provide benefits to research and do not necessarily mean that the trial will always be cheaper to run than non-ADs;
3. Become willing to accept that some aspects of supporting an AD may be more resource-intensive than with traditional trials, particularly as units build their experience in running these trials;
4. Consider ways to allow more flexibility in specifying resources required by ADs, including more space in application forms to describe how resources are impacted by adaptations, and space for multiple funding estimates;
5. Consider supporting more methodology research that could investigate reducing this additional cost (e.g., through Studies Within A Trial which are currently funded in National Institute for Health Research (NIHR, UK) trials [37]);
6. Introduce more funding for shared infrastructure (e.g., platform trial infrastructure and innovative design advice) for developing and efficiently delivering innovative trials;
7. Have more cross-panel and cross-funder opportunities for funding seamless trials and master protocols rather than operating in fixed phases of trials;
8. Consider appropriate funding mechanisms for dealing with changes to trial costings due to adaptation;
9. Avoid financially penalizing organisations for the efficiency achieved in studies stopped early by allowing flexible use of the saved resources (e.g., to cover the cost for the development of subsequent investigations).

We would also advise funders, in collaboration with applicants, to consider whether overly complex ADs could be simplified (with resource savings) without loss of benefit [11]. Conversely, funders should encourage applicants to add adaptive elements if they would be beneficial to information or patients.

We would like to highlight that several funders have made great progress in addressing barriers to the wider use of innovative designs. It is also understandable that some funders operate under considerable constraints (e.g. annual budgets that cannot be carried forward) that make it more difficult to address some barriers.

## Discussion

Once there is a compelling reason that an AD brings better and quicker evidence, resourcing and justifying it is a time-consuming process. Our five-step approach outlined here provides structure to the process. This complements literature covering the process of designing adaptive trials such as [3, 4, 38], addresses barriers raised in the implementation of ADs [39, 40], resourcing of clinical trials [41–43] and investigation of additional resources required to support adaptive trials [7, 8].

We have restricted attention to resourcing the trial after it is funded. Like any clinical trial, the process of designing an adaptive trial requires a substantial amount of input from a multidisciplinary team. However, more specialist expertise and a greater amount of time is generally required for an AD. This is difficult to resource other than from institutional core funding for trial development infrastructure. Some funders may offer development grants which would help cover this additional work upfront.

We would emphasise that ADs can provide many benefits that may outweigh cost considerations. These include lower average time taken to complete the trial, better outcomes for participants recruited to the trial, and higher-quality evidence provided by the trial. In addition, the apparent higher costs of supporting an AD may just be a ‘worst-case-scenario’ and be offset by substantially reduced costs if the trial finishes early. The only work we are aware of that investigated differences in cost between adaptive and non-adaptive trials in practice is Martin et al. [8], which investigated several different cost-drivers using regression models. The authors did not find a significant cost difference between adaptive and non-adaptive designs although it is likely to be difficult to estimate reliably as AD use may be different by phase and indication.

The key factor that justifies an AD is the ratio of benefit it provides (both to the efficiency and patient benefit of the trial itself and the long-run impact of the evidence generated) to the additional cost it incurs [44, 45]. Research that can inform and improve both parts of this ratio is needed. Trial methods that can maintain or improve the speed and quality of interim analyses whilst reducing the resources required would be very useful; methods that may improve the benefit provided without requiring more resources would similarly be of great interest. Overall, a framework for better quantifying the benefit of an AD in the presence of real-world issues such as delay in assessment of outcomes [11] would help justify this to funders.

ADs provide advantages and complexities for other types of analyses too. In our CAT research, and this guidance, we have not highlighted areas such as health economics, qualitative research and evidence synthesis. Previous work, e.g. Flight et al. [32] has noted the impact of ADs on health economic considerations. However, further work is needed for investigating how ADs affect the resources required for health economists and other methodological disciplines. Additional resources may be required for the design of the adaptive trial, contributing to interim decision making and appropriately analysing the final data to account for the AD.

As the recommendations in this paper are based on investigation of UK academic CTUs, we acknowledge they are most relevant to academic trials run in the UK. Co-authors of this paper have experience with international academic funders including the European Commission, the Deutsche Forschungsgeneinschaft (DFG, Germany), National Institutes of Health (NIH, USA), National Health and Medical Research Council (NHMRC, Australia) and Fight Kids Cancer (Belgium) suggesting that the issues identified here are very similar. With trials funded by large international pharmaceutical companies, some of the guidance will be relevant but it is likely that there is much more of a focus on average cost, power of trials, and portfolio optimisation. We would welcome further papers that consider how our recommendations may be best tailored to fit trials outside of the UK and run by industry including smaller companies.

The evolution of application forms by funders that would enable a rationale for costs to sit alongside the design choices, paired with greater flexibility in the way costs are presented to funders, could improve transparency whilst enabling the many benefits of innovation in trial design to be realised more broadly in clinical research through the funding of more trials using ADs. It is also important that innovation in trial designs that can lead to improved efficiency, quality of evidence, and patient benefit are incentivised by funders.

Although we have concentrated on ADs, the process could be used for other innovative approaches. For example, master protocols [9] (including basket trials and umbrella trials, and platform and living protocols) are not necessarily adaptive but may require similar considerations of appropriately resourcing them. Platform trials, which offer the opportunity to add in new arms, may require considering the additional costs incurred from the additional arm compared with the fixed costs of the platform.

Through better guidance on appropriately resourcing ADs, we hope that their use can continue to increase, which will play an important role in improving patient outcomes and improving research productivity.

## Supplementary Information

**Additional file 1.** Brief overview of each scenario used in the Costing Adaptive Trials mock costing exercise.

**Additional file 2.** Costing spreadsheet.

## Data Availability

Data sharing is not applicable to this article as no datasets were generated or analysed during the current study.

## Abbreviations

AD: Adaptive design
CAT: Costing Adaptive Trials
CDMS: Clinical Data Management System
CRC: Clinical Research Collaboration
CTIMP: Clinical Trial of Investigational Medicinal Product
CTU: Clinical Trials Unit
DFG: Deutsche Forschungsgemeinschaft
DPFS: Developmental Pathway Funding Scheme
HEAP: Health Economic Analysis Plan
IMP: Investigational Medicinal Product
MAMS: Multi-arm multi-stage
MRC: Medical Research Council
NHMRC: National Health and Medical Research Council
NHS: National Health Service
NIH: National Institutes of Health
NIHR: National Institute for Health Research
PIS: Patient Information Sheet
SAP: Statistical analysis plan
SOP: Standard operating procedure
VOIA: Value of information analysis
