# Supplementary material for: Practical guidance for planning resources required to support publicly-funded adaptive clinical trials
Source: BMC Med. 2022 Aug 10;20:254. doi: 10.1186/s12916-022-02445-7 (PMC9364623; doi:10.1186/s12916-022-02445-7)
Supplement: Supplementary file 1 — Additional file 1. Brief overview of each scenario used in the Costing Adaptive Trials mock costing exercise. [file 12916_2022_2445_MOESM1_ESM.docx]

| **Scenario** | **Adaptive design** |
| --- | --- |
| Two-arm randomised controlled trial assessing addition of biomarker-testing to an existing early warning score in the management of patients with suspected sepsis in the emergency department | Group-sequential design |
| A randomised dose-finding study of JAK1 inhibitor for patients with active rheumatoid arthritis | Design that updates dose allocation in second stage based on optimal design fitted to stage 1 patients. |
| A multi-arm open-label phase 3 trial comparing regimens for treating intermediate and high-risk oropharyngeal cancer | Multi-arm multi-stage design with early stopping for lack of benefit. |
| A randomised controlled trial assessing clinical and cost-effectiveness of earlier treatment of ovarian hyper-stimulation syndrome | Adaptive umbrella study, allowing early stopping of arms within patient subgroups. |
| Randomised double-blinded placebo-controlled trial of the efficacy of nicotinic acid derivative (NAD) for treatment of fatigue in mitochondrial disease | Sample size re-assessment |

Supplementary Table 1 – Brief overview of each scenario used in the Costing Adaptive Trials mock costing exercise
